# A Comprehensive Review of Catheter-Related Thrombosis

**DOI:** 10.3390/jcm13247818

**Published:** 2024-12-21

**Authors:** Marina López-Rubio, Marta-Olimpia Lago-Rodríguez, Lucía Ordieres-Ortega, Crhistian-Mario Oblitas, Sergio Moragón-Ledesma, Rubén Alonso-Beato, Luis-Antonio Alvarez-Sala-Walther, Francisco Galeano-Valle

**Affiliations:** 1 Venous Thromboembolism Unit, Internal Medicine Department, Hospital General Universitario Gregorio Marañón, 28007 Madrid, Spain; marinalopezrubio@outlook.com (M.L.-R.); lucia.oomere@gmail.com (L.O.-O.); crhistian.cao@gmail.com (C.-M.O.); smoragonledesma@gmail.com (S.M.-L.); ralonso_92@hotmail.com (R.A.-B.); laalvarez@med.ucm.es (L.-A.A.-S.-W.); paco.galeano.valle@gmail.com (F.G.-V.); 2 School of Medicine, Universidad Complutense de Madrid, 28040 Madrid, Spain; 3 Sanitary Research Institute Gregorio Marañón, 28009 Madrid, Spain

**Keywords:** catheter-associated venous thrombosis, hemodialysis catheter-associated thrombosis, pacemaker-related thrombosis, upper extremity deep vein thrombosis, venous thromboembolism

## Abstract

Catheter-related thrombosis (CRT) is a frequent and potentially serious complication associated with the widespread use of intravascular devices such as central venous catheters, including peripherally inserted central catheters and implantable port systems, pacemakers or implantable cardioverter-defibrillators. Although CRT management has been informed by guidelines extrapolated from lower extremity deep vein thrombosis (DVT), unique challenges remain due to the distinct anatomical, pathophysiological, and clinical characteristics of upper extremity DVT. Risk factors for CRT are multifactorial, encompassing patient-related characteristics such as cancer, prior venous thromboembolism, and infection, as well as catheter-specific factors like device type, lumens, and insertion site. The diagnosis of CRT relies primarily on ultrasonography; however, computed tomography angiography and magnetic resonance imaging play a complementary role, particularly in anatomically challenging cases or when complications such as pulmonary embolism or superior vena cava syndrome are suspected. Treatment strategies for CRT include anticoagulation, catheter removal when feasible, and, in select cases, local thrombolysis or catheter-directed interventions. Anticoagulation remains the cornerstone of therapy, with direct oral anticoagulants increasingly favored due to their safety profile and efficacy. This article provides a detailed review of CRT, focusing on clinical features, diagnostic methods, and treatment strategies while addressing specific challenges in managing pacemaker and hemodialysis catheter-related thrombosis.

## 1. Introduction

The superficial venous system of the upper extremities consists of the basilic, cephalic, median cubital, and accessory cephalic veins. Among these, the basilic and cephalic veins are the most commonly utilized to place peripheral venous catheters and peripherally inserted central catheters (PICCs). Although the basilic vein is part of the superficial system for most of its course, it transitions to the deep venous system as it pierces the brachial fascia at mid-arm and continues until it joins the brachial vein [1].

The deep venous system of the arm includes the brachial veins and the axillary vein, which becomes the subclavian vein after crossing the first rib. Upper extremity deep vein thrombosis (UEDVT) is a relatively uncommon entity within venous thromboembolism (VTE), involving thrombosis in the arm veins [1,2]. In some cases, it is associated with more severe conditions, such as pulmonary embolism (PE) or superior vena cava syndrome (SVCS) [3,4]

**Figure 1:**
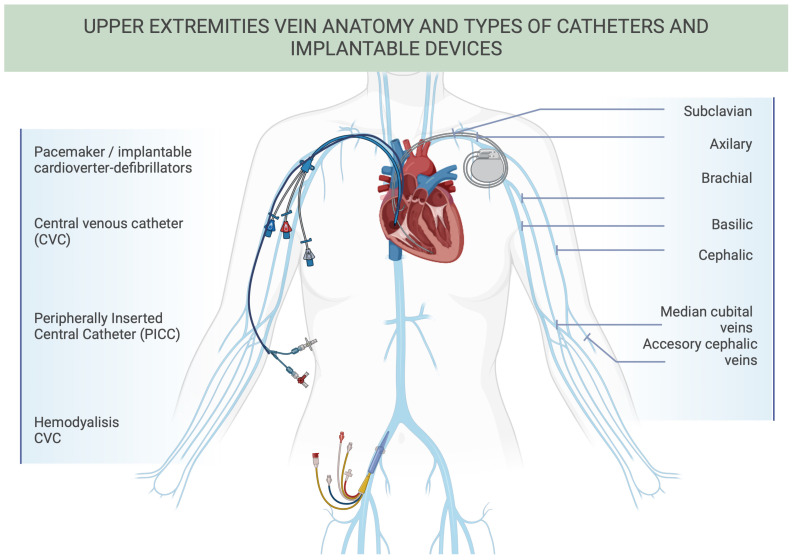
Upper extremities vein anatomy and types of catheters and implantable devices. Visual representation of the venous anatomy of the upper extremities, illustrating the subclavian, axillary, brachial, basilic, cephalic, median cubital, and accessory cephalic veins. The figure also highlights the placement and types of commonly used catheters, including central venous catheters (light blue), peripherally inserted central catheters (dark blue), hemodialysis catheters (yellow), and implantable devices such as pacemakers or implantable cardioverter-defibrillators (gray).

Catheter-related thrombosis (CRT) is classified as a secondary UEDVT, accounting for 80–90% of these cases [5,6]. This review discusses different types of catheters, which are detailed below. Central venous catheters (CVCs) can be placed via subclavian, jugular, or femoral access points; PICCs terminate at the superior vena cava-right atrium junction; and midline catheters terminate in large-caliber peripheral veins such as the proximal basilic or cephalic vein [3,7]. Implantable devices, among which are pacemakers (PMs) and implantable cardioverter-defibrillators (ICDs), as well as CVCs used for hemodialysis, are also reviewed (Figure 1).

The management of CRT poses distinct challenges compared to other forms of venous thrombosis, particularly in complex clinical scenarios. Current guidelines provide limited evidence and low-grade recommendations due to the heterogeneity and scarcity of robust studies in this area [8,9,10,11,12]. This gap underscores the need for further research to establish standardized approaches for diagnosis and treatment.

This article provides a comprehensive review of the clinical manifestations, diagnostic approaches, and therapeutic strategies for CRT, emphasizing recent advances and ongoing controversies in the field. Additionally, it addresses specific conditions including PM-related thrombosis and hemodialysis catheter-associated thrombosis, highlighting the unique challenges and management strategies required for these patient populations.

## 2. Central Venous Catheters, Peripherally Inserted Central Catheters, and Implantable Port Systems

### 2.1. Epidemiology

UEDVT accounts for approximately 5% of all limb cases of deep vein thrombosis (DVT). Incidence rates vary widely across global registries, likely due to differences in the included populations. In 2017, Cote et al. [13], using data from the RIETE registry, reported an incidence of UEDVT ranging between 4% and 10%. Similarly, Yamashita et al. [14], from the COMMAND-VTE registry, and Ageno et al. [5], from GARFIELD-VTE Registry, documented incidences between 1% and 6%.

The vast majority of UEDVT cases are secondary events, constituting 80–90% of these cases, and are frequently associated with intravascular devices such as CVCs, PICCs, PMs, or implantable port systems [5,6]. One study reported up to a sevenfold increase in UEDVT risk in the presence of a CVC (adjusted odds ratio [OR] 7.3; 95% CI: 5.79–9.21; *p* < 0.0001) [15].

The overall annual incidence of CRT is estimated to be 0.4 to 1.0 per 10,000 individuals. This incidence appears to have increased in recent years, likely due to the rising use of catheters and the growing number of oncology patients [6,15,16,17].

### 2.2. Clinical Manifestations

CRT presents a wide spectrum of clinical manifestations, which vary significantly among studies due to differences in diagnostic criteria and methodologies.

Importantly, a substantial proportion of patients remain asymptomatic. Zochios et al. [18], in their investigation of PICC-related thrombosis in critically ill patients, observed that up to 50% of all cases were asymptomatic. However, the true proportion might be even higher, as many cases go undiagnosed. This underscores the necessity for a raised index of suspicion, particularly in high-risk patients, to ensure timely recognition and management [19].

The incidence of symptomatic CRT varies widely among studies, largely influenced by the diagnostic criteria and imaging modalities employed. Rajasekhar et al. [20] reported symptomatic CRT incidence rates of 1% to 5%, while Baskin et al. [21] described higher rates, reaching up to 28% in adults and 12% in pediatric patients with CVCs.

CRT can sometimes manifest solely as catheter malfunction [20]. Catheter occlusion can be partial, preventing aspiration but allowing infusion, or complete, characterized by an inability to aspirate or infuse. This complication occurs in 14–36% of patients within the first two years after catheter placement [3,21]. Notably, these functional issues are not always linked to CRT. However, in cancer patients with catheter dysfunction, up to 25% were found to have underlying thrombosis [22]. Malfunction may also be accompanied by fever, particularly when septic thrombophlebitis progresses to CRT [20].

The clinical manifestations of CRT otherwise resemble those of UEDVT. Common symptoms include venous distension (reported in up to 100% of cases), arm swelling (93%), and blue discoloration (77%) [7,23].

PE occurs less frequently in UEDVT than in lower extremity DVT (LEDVT), with incidence rates ranging from 3% to 12% [3]. Data from the RIETE registry showed symptomatic PE occurred in 9.8% of patients with UEDVT, compared to 25% in those with LEDVT [13]. Interestingly, Cote et al. found that PE incidence was higher in patients with CRT compared to those with primary UEDVT. However, multivariable analysis suggests that this association might be influenced by confounding variables, particularly cancer [13]. Other studies have similarly indicated that PE is more common in critically ill patients with UEDVT, in addition to those with malignancy [24,25].

SVCS is a well-documented complication of CRT [26]. Intravascular devices, like catheters or PMs, account for approximately 28% of all SVCS cases, making CRT the leading non-malignant cause of this syndrome [4].

Although less common, catheter related atrial thrombosis (CRAT) may occur. CRAT is potentially life-threatening, as it can result in PE, systemic embolisms, thrombus infection, or hemodynamic compromise, depending on thrombi size [27].

The incidence of post-thrombotic syndrome (PTS) following CRT varies between studies, with reported rates ranging from 7% to 46%. While some authors suggest that PTS is more common in CRT cases, others argue that its incidence may be lower compared to primary thrombosis. This discrepancy likely arises from variations in diagnostic methodologies and patient populations. Notably, Elman et al. [28], in a systematic review, found that the incidence of PTS following CRT was lower than in primary thrombosis, particularly in cases involving PICCs.

### 2.3. Risk Factors

**Figure 2:**
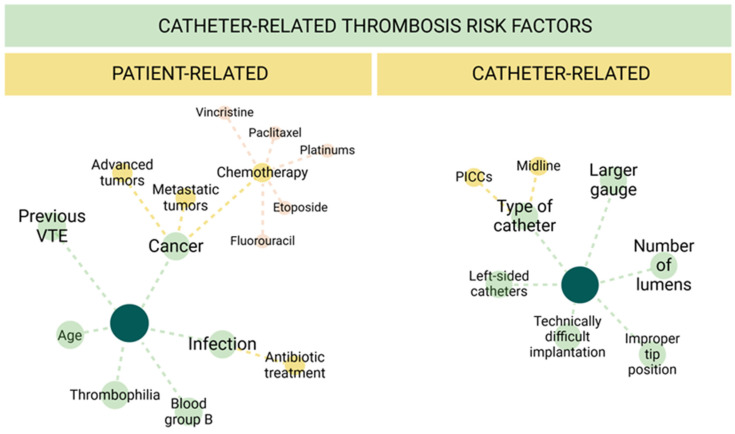
Catheter-related thrombosis risk factors. Overview of the main patient- and catheter-related risk factors associated with CRT. VTE (venous thromboembolism), PICCs (peripherally inserted central catheters).

Unlike classical VTE, where risk factors are well-defined, the factors associated to CRT remain incompletely understood. Several studies have explored this topic, but most have limitations such as being retrospective, having small sample sizes, or focusing on highly specific patient populations. These issues have contributed to ongoing controversies regarding the role of various risk factors in CRT development. Broadly, risk factors can be categorized into those related to patient characteristics and those associated with the catheter or its insertion [29] (Figure 2).

#### 2.3.1. Patient-Related Risk Factors

Among patient characteristics, a previous history of VTE appears to be one of the main risk factors for CRT [19], as confirmed by various meta-analyses [30,31] and observational studies [32,33,34,35,36]. Liu et al. [31] analyzed data from 2874 patients across 12 studies, including 439 CRT cases, and found that prior VTE had the highest impact on CRT risk, with an OR of 3.75 (95% CI 1.02–13.85). Similarly, Saber et al. [30] conducted a meta-analysis on 12 studies involving 5636 oncology patients, of whom 425 had CRT. The only patient-related factor they identified as increasing CRT risk was a past history of DVT, with an OR of 2.03 (95% CI 1.05–3.92).

Another widely recognized risk factor for CRT is cancer, with an incidence of symptomatic PICC-related UEDVT ranging between 2.7% and 13.8% among cancer patients following PICC insertion [19,37,38]. Cancer contributes to CRT through varied mechanisms, including the release of procoagulant factors, direct vein compression, or cancer-related factors like surgery, bedrest, or pro-thrombotic treatments [31,39,40,41]. Studies have reported a 1.7-to-2-fold increase in CRT among cancer patients compared to non-cancer patients [31,34].

Efforts have been made to identify cancer patients at a higher CRT risk to better target those who might benefit from prophylactic anticoagulation. Ma et al. [41] performed a meta-analysis of 19,824 oncology patients, demonstrating that advanced tumors and metastasis significantly increase the risk of CRT, with ORs of 3.08 (95% CI 1.93–4.91) and 3.06 (95% CI 1.9–4.91), respectively. This finding is further supported by other studies [42,43]. The same meta-analysis [41] also found that chemotherapy was associated with a higher CRT incidence (OR 1.96, 95% CI 1.27–3.02), a relationship later corroborated by several other authors [34,44,45,46]. Chemotherapy may contribute to CRT through different mechanisms, comprising direct endothelial damage, acidosis, and adverse reactions leading to increased bed rest and immobilization, thus exacerbating hypercoagulability [41]. Specific chemotherapy agents associated with a higher CRT risk include fluorouracil [41,47], etoposide [41,42], platinum-based drugs [37,41], paclitaxel [41], and vincristine [37].

The risk of CRT may also vary by cancer site or histology, although no consensus has been reached. Higher risks have been reported for gastrointestinal [37], hematological [41], head and neck [20], and gynecological [22,41] cancers. These differences likely reflect patient heterogeneity and confounding factors, such as variations in chemotherapy regimens, which can influence CRT development.

Infection is another significant patient-related risk factor for CRT that is associated with an approximately two-fold increase in risk, although not all authors report statistical significance [31,37,48]. Inflammatory responses during infections may promote CRT through mechanisms such as acidosis, endothelial damage, and disbalance in the hemostatic system and platelet activity [48]. In fact, Engelmann et al. [49] coined the term immunothrombosis, and explained that innate immune cells can become prothrombotic and lead to the formation of intravacular microthrombi. Although the role of immunothrombosis in CRT is not clear, some studies have found a relation between systemic immune inflammation indexes (such as platelet-to-lymphocyte ratio) and PICC-related DVT [50].

Additionally, some studies suggest that antibiotic use correlates with an increased CRT risk [51], potentially due to both the underlying infection and the direct effect of certain antibiotics. Vancomycin at high doses has been suggested to increase the risk of CRT due to its potential toxicity to vessel walls [51,52]. Amphotericin B has also been associated with a higher CRT risk [32].

Other patient characteristics have shown associations with CRT, although less consistent. CRT appears to be more frequent in older patients [37,48,53]. Incidence rates increase from 0.032 per 1000 individuals among younger adults (aged 20–39 years) to nearly 0.297 per 1000 in those aged 75 years or older [17]. Additional proposed risk factors include hypertension, chronic kidney disease [51,54,55], and blood group B compared to group 0 [45].

Regarding thrombophilia, a meta-analysis by Dentali et al. concluded Factor V Leiden and the G20210A prothrombin mutation are associated with a 4- to 5-fold higher CRT risk in cancer patients, although the studies did not specify whether mutations were present in homozygosis or heterozygosis [56].

As for sex, most studies report a higher CRT risk in males [37,52]. However, a retrospective study of 497 patients with PICCs found a higher CRT incidence in females, potentially due to the smaller caliber of veins in women, leading to catheters occupying a larger proportion of the venous lumen [57].

The relationship between diabetes and CRT remains controversial. Diabetes is often considered a risk factor due to hyperglycemia-induced changes in blood flow and microcirculation [43,48]. Nonetheless, a retrospective cohort study suggested diabetes might act as a protective factor, though the underlying mechanism remains unclear and warrants further investigation [54].

#### 2.3.2. Catheter-Related Risk Factors

The main risk factor consistently identified across studies is the type of catheter, with CRT being approximately 2 to 3 times more frequent in PICCs compared to CVCs. The reported incidence of PICC-associated thrombosis is 2% in the general population, increasing up to 13.8% in cancer patients, as previously mentioned. The incidence of CRT with midlines (peripheral access devices) is similar to that of PICCs [7,37]. For CVCs, the incidence varies across studies, ranging from 1.4% to 15.5%, depending on the setting and patients’ comorbidities [24,58]. CRT associated with implantable ports is the least common among these devices, with incidences reported between 1% and 3.8% in oncological patients [30,59,60,61,62,63,64,65,66,67]. This discrepancy may be attributed to PICCs being inserted into smaller-diameter veins and having longer catheters, both of which increase contact between the catheter and the vascular wall, leading to endothelial damage and blood flow reduction [60].

Other catheter characteristics that influence CRT risk include the catheter gauge and the number of lumens. Larger devices are more likely to cause endothelial damage [31,33,34,45,51,57,61,62,68]. Song et al. conducted a retrospective study of 549 hospitalized patients aged 65 or older who had PICC insertions. They found both catheter size and the number of lumens to be independent risk factors for CRT development. Specifically, for catheter calibers, the OR was 1.82 (95% CI 1.25–2.54) for 5-Fr and 3.86 (95% CI 1.37–8.25) for 6-Fr catheters compared to 4-Fr catheters. Regarding the number of lumens, the OR was 1.93 (95% CI 1.36–2.57) for double-lumen catheters and 3.76 (95% CI 1.67–5.93) for triple-lumen catheters [33]. Other studies confirm these findings, with CRT risk approximately doubling for 5-Fr catheters and tripling for 6-Fr catheters [33,44,45]. Similarly, catheters with two lumens were associated with a 1.6- to 2.5-fold increase in CRT risk, while those with three lumens showed a 2- to 3.76-fold increase, depending on the study. Interestingly, the number of lumens was identified as an independent risk factor in multiple studies, suggesting that increased risk is not solely due to the larger gauge of multi-lumen catheters [31,33,34,44].

Improper positioning of the catheter tip is also associated with a higher risk of CRT. Positioning the tip at the junction of superior vena cava (SVC) and right atrium appears to be protective, likely due to reduced direct contact with the endothelium [30,52,69].

Technically challenging procedures or multiple insertion attempts are linked to an increased risk of CRT [47,68,69,70]. Additionally, some studies report up to a twofold increase in CRT risk with left-sided catheters [51,61,68], though large meta-analyses have failed to confirm this association [30,31].

The influence of catheter insertion remains controversial. Some studies suggest higher risks with subclavian vein insertion [30], while others report greater risks after basilic [51] or cephalic vein [61] placement.

Other risk factors mentioned in a few studies, and thus with weaker evidence, include the use of open-ended catheters compared to those with valves (probably due to reduced reflux and turbulent blood flow), presence of a prior CVC, catheter placement during surgery, catheter dwell time, and catheter material. Regarding material, conflicting evidence exists as to whether silicone is less thrombogenic than polyurethane [37,48,57,68,70].

### 2.4. Diagnosis

The cornerstone of CRT diagnosis is Doppler ultrasonography (US), which has replaced venography as the gold standard for diagnosing VTE in both upper and lower extremities. Doppler US offers a sensitivity of 91% and a specificity of 93% [71], though results can vary slightly between studies [72]. Specifically for CRT, a systematic review of five clinical trials involving 96 patients reported a sensitivity ranging from 56% to 100% and a specificity of 94% to 100% [73]. Additionally, a prospective study of 18 patients with malignancy and CRT found that the US achieved 82% sensitivity and 82% specificity when compared to venographically confirmed DVT [74].

Doppler US is simple, cost-effective, and non-invasive [3]. However, it presents challenges in certain cases due to anatomical factors, particularly the clavicle, which can obstruct visualization and compression of the mid-portion of the subclavian vein. This limitation can lead to false-negative results [71]. Diagnosis of CRT via US relies on identifying thrombi within the vein, absence of venous compressibility, or lack of Doppler flow [19].

Computed tomography angiography (CTA) and magnetic resonance imaging (MRI) are valid diagnostic alternatives in cases of high suspicion and negative US findings, particularly for certain anatomical locations [3]. However, no specific studies directly compare these imaging modalities to Doppler US in CRT patients [75,76]. MRI, due to its lack of radiation, is particularly valuable in the pediatric population [77].

Fallouh et al. [19] propose a diagnostic algorithm for CRT in patients with PICCs based on clinical probability and US findings. In cases of persistent high clinical suspicion despite negative US results, additional imaging with venography, CTA, or MRI is recommended. If the clinical probability is low, observation and repeat US may be appropriate [19].

The role of D-dimer as a diagnostic marker remains controversial. While it demonstrates high sensitivity (100%), its specificity is very low (14%) [78]. D-dimer levels may be lower in CRT compared to other thrombotic conditions, as is often observed in UEDVT and other atypical VTE locations [79]. A recent study found that patients with low D-dimer (<500 ng/mL) were more likely to have UEDVT than those with elevated D-dimer [80]. However, D-dimer levels in CRT can also be influenced by confounding factors such as cancer or infection [74]. Emerging biomarkers like P-selectin have shown potential usefulness for diagnostic applications [81], either alone or combined with clinical scores (Wells’ score) [82]. However, in the setting of short-term prognosis for PE, soluble P-selectin has shown poor results [83].

The CONSTANS algorithm, validated for UEDVT, includes catheter presence as one of the criteria to diagnose UEDVT, along with limb pain, unilateral edema, and the absence of an alternative diagnosis. When two or three criteria are present, a US is indicated to confirm DVT. If negative, D-dimer testing is necessary. Elevated D-dimer levels require repeated US, while normal levels rule out DVT. If less than two criteria in the CONSTANS score are met, D-dimer levels should be determined first. If elevated, a US should be performed, whilst if negative, DVT can be ruled out [7]. Additionally, specific algorithms have been proposed for CRT in oncologic patients [84]. Among cancer patients, scores such as Khorana, PROTECHT, and COMPASS have shown a strong positive association with CRT development [59].

Doppler US remains the primary diagnostic tool for CRT due to its high sensitivity and specificity, despite certain limitations that may result in false negatives. Clinical scores and biomarkers can provide additional support in the diagnostic process. In cases of diagnostic uncertainty or suspected false negatives, follow-up US or alternative imaging modalities should be considered, such as CTA, MRI, or venography.

### 2.5. Treatment

**Figure 3:**
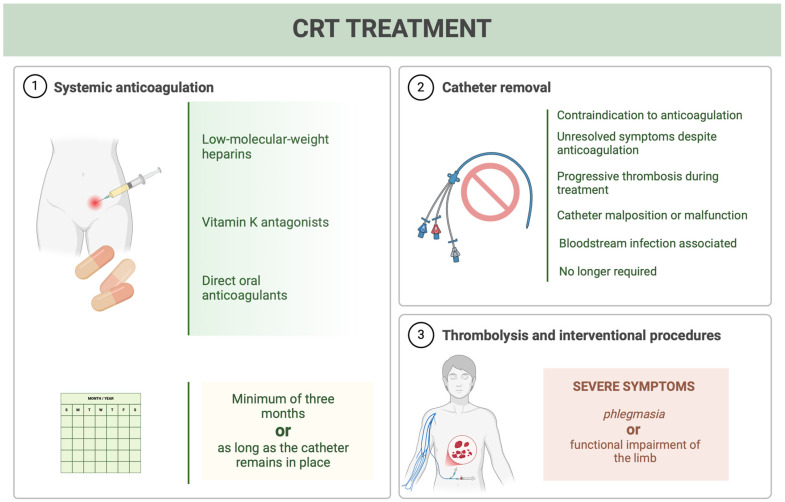
Catheter-related thrombosis treatment. Overview of catheter-related thrombosis (CRT) treatment strategies. (1) Systemic anticoagulation, (2) Catheter removal, (3) Thrombolysis and interventional procedure.

CRT treatment involves three key approaches: systemic anticoagulation, catheter removal, and thrombolysis or interventional procedures for selected cases [19] (Figure 3).

#### 2.5.1. Anticoagulation

There is a lack of randomized clinical trials (RCTs) specifically addressing anticoagulant selection or treatment duration in CRT. Most recommendations are extrapolated from studies on LEDVT, where evidence is more robust [85]. In the context of UEDVT, most studies focus on cancer patients, leaving limited data for non-cancer populations.

Low-molecular-weight heparins (LMWHs) such as enoxaparin, or fondaparinux, are generally preferred over unfractionated heparin due to their favorable safety profile and ease of use [86,87]. Vitamin K antagonists (VKAs) remain an acceptable alternative for non-cancer patients or for those in whom LMWH is contraindicated [19]. The European Society of Vascular Surgery (ESVS) guidelines from 2021 recommend LMWH alone or followed by VKA with a grade 2a recommendation [88,89].

Direct oral anticoagulants (DOACs) have gained acceptance in the management of CRT. Observational studies support their safety and efficacy, with a high rate of catheter function preservation [90].

- Rivaroxaban: In the CATHETER 2 study, 70 cancer patients were treated with rivaroxaban (15 mg twice daily for 21 days, then 20 mg once daily). Thrombosis
- Progression was prevented in 96% of patients, while major and minor bleeding occurred in 5.7% and 8.6%, respectively [91].
- Apixaban: The CATHETER 3 study included 87 cancer patients treated with apixaban (10 mg twice daily for 7 days, then 5 mg twice daily). Thrombosis resolution or stabilization was achieved in 92% of cases. Major bleeding occurred in 3.4%, minor bleeding in 10.3%, and overall mortality was 8%, although no deaths were related to treatment [92].
- Edoxaban: A study by Porfidia et al. compared edoxaban, enoxaparin, and fondaparinux in 74 women with gynecologic or breast cancer and CRT. Residual thrombosis rates were low (4.5% for edoxaban, 4.8% for enoxaparin, and 16.0% for fondaparinux). Recurrence was negligible across groups, and no major bleeding events were reported. However, clinically relevant bleeding occurred in 9.5% of patients treated with enoxaparin and 3.2% with fondaparinux. Catheter function was preserved in all cases [93].

Comparative studies suggest that DOACs achieve similar outcomes to LMWH or VKA in terms of recurrence, bleeding, and mortality [93,94].

Current guidelines recommend a minimum anticoagulation duration of three months or for as long as the catheter remains in place. However, the decision should be individualized based on the patient’s risk of thrombosis and bleeding [8,9,10,11,12].

Data from the RIETE registry, one of the largest cohorts of CRT patients, showed an annual recurrence rate of approximately 1.5% after discontinuation of anticoagulation. Extended anticoagulation beyond three months or for patients with transient risk factors significantly reduced the risk of thrombotic recurrence. These findings emphasize the importance of tailoring anticoagulation duration to individual patient risk and clinical context, balancing the benefits of preventing recurrence with the risk of bleeding complications [95].

#### 2.5.2. Catheter Removal

Since the presence of a catheter represents a persistent thrombogenic risk factor, its removal should be considered under specific circumstances. These include contraindications to anticoagulation, unresolved symptoms despite anticoagulation therapy, progressive thrombosis during treatment, suspicion of catheter malposition or malfunction, suspected bloodstream infection associated with the catheter, and cases where the catheter is no longer required [8,9,10,12]

In contrast, maintaining the catheter is feasible while anticoagulation therapy is administered. Studies in cancer patients with CRT indicate that catheters can be maintained without complications related to thrombosis [10,86,91,92]. Kovacs et al. [86] conducted a study involving cancer patients with CRT treated with either LMWH or VKA for 12 weeks and found that none required catheter removal due to thrombosis. Comparable evidence supports the use of DOACs as an effective anticoagulation option without requiring catheter removal [91,92].

#### 2.5.3. Thrombolysis and Interventional Procedures

Thrombolysis and interventional procedures represent targeted therapeutic strategies for selected patients with CRT. Observational data suggest that early implementation of thrombolytic therapy, in conjunction with anticoagulation, can improve venous patency in the upper extremities despite an elevated risk of bleeding [19].

Thrombolysis is typically reserved for patients with severe symptoms, such as phlegmasia or functional impairment of the limb; extensive thrombus burden in the subclavian or axillary veins; symptom duration under 14 days; good functional status; and an expected life expectancy of at least one year and low bleeding risk [19].

Sabeti et al. analyzed 50 patients with subclavian-axillary thrombosis, comparing anticoagulation alone to systemic thrombolysis. The thrombolysis group showed a higher rate of complete recanalization (44% vs. 20% in the anticoagulation-only group). However, the thrombolysis group also experienced a notable incidence of major bleeding (12%) [96].

Recent trends favor catheter-directed thrombolysis (CDT) over systemic thrombolysis, given its potential for similar efficacy with a reduced rate of adverse events. Maleux et al. assessed the efficacy and safety of CDT in patients with UEDVT, both with and without cancer. This study reported complete recanalization in 88% of patients and partial recanalization in 12%, accompanied by significant improvement in symptoms such as pain and edema. Major bleeding occurred in 11% of patients, highlighting a persistent risk associated with the procedure [97].

These findings, along with evidence extrapolated from studies on LEDVT, suggest that thrombolysis and CDT may be viable strategies for highly selected patients. While these treatments demonstrate high rates of recanalization, they also carry a substantial risk of major bleeding [96,97,98,99,100,101,102].

Given this balance of risks and benefits, a 2015 narrative review on CRT proposed an algorithm advocating for thrombolysis or interventional procedures as first-line treatment only in cases of phlegmasia or functional compromise [19].

### 2.6. Prevention

Given the incidence and clinical impact of CRT, the question has been raised as to whether patients should receive thromboprophylaxis after implantation of a central venous access. The first proposed measure to minimize CRT is to carefully assess the indication for vascular access, along with selecting the most appropriate catheter type after evaluating the patient’s risk of thrombosis and bleeding [19].

Pharmacological prophylaxis has been evaluated in multiple studies, primarily in oncological patients [103]. Several Cochrane meta-analysis have been conducted on this topic. The most recent one, in 2018, analyzed 13 RCTs to examine the efficacy and safety of prophylactic-dose heparin or low-dose VKA in cancer patients with CVCs. The study found that LMWH likely reduces the incidence of symptomatic CRT compared to no LMWH, with no differences in three-month mortality or major bleeding. No beneficial or detrimental effects were reported for VKA versus no VKA, nor were significant differences found between LMWH and VKA [104].

Conversely, D’Ambrosio et al. [105] performed a meta-analysis with 12 RCTs comparing thromboprophylaxis with placebo, reporting a significant reduction in symptomatic VTE. More recently, Li et al. conducted a meta-analysis including 12 RCTs evaluating thromboprophylaxis with either LMWH, VKA, or DOACs in cancer patients with CVCs. They found a lower incidence of VTE in patients who received prophylaxis compared to those who did not, with no difference in major bleeding or clinically relevant non-major bleeding but with an increase in minor bleeding [106].

The AVERT trial, an RCT evaluating apixaban 2.5 mg twice daily for primary thromboprophylaxis in ambulatory cancer patients, included 217 patients who had CVCs and initiated chemotherapy. A subgroup analysis showed a significant reduction in VTE (18.7% in the placebo group versus 4.8% in the apixaban group), with no differences in major bleeding [107]. However, another RCT evaluating rivaroxaban 10 mg daily against placebo for primary prophylaxis in cancer patients with newly inserted CVCs reported no differences in VTE [108].

Recently, Pfeffer et al. published the results of a clinical trial involving 22 ambulatory cancer patients undergoing central line placement, evaluating the safety and efficacy of *gruticibart*, an anti-factor XI monoclonal antibody. They found no significant adverse events or bleeding, and a lower incidence of CRT on surveillance ultrasound performed two weeks after insertion [109]. Lastly, an RCT evaluated the administration of aspirin (100 mg) versus placebo in 481 patients with malignant tumors receiving chemotherapy who underwent PICC insertion. The analysis revealed a lower incidence of CRT in the aspirin group, with no bleeding events [110].

Currently, clinical guidelines do not recommend routine pharmacological thromboprophylaxis for ambulatory cancer patients with CVCs [11,90,111]. The European Society of Medical Oncology recommends assessing thrombotic risk using various scores and considering thromboprophylaxis in high-risk patients, regardless of the presence of a CVC [11].

## 3. Intracardiac Devices

In contemporary clinical practice, PMs and ICDs are being increasingly utilized. Their use can lead to complications, including PM lead migration, infection, and post-implantation venous thrombosis or occlusion [112].

### 3.1. Epidemiology and Pathophysiology

The incidence of UEDVT associated with intracardiac devices varies substantially, ranging from 0.5% to 30% of all implanted devices across different studies [112,113,114,115]. These differences are likely due to varying diagnostic criteria and, most importantly, whether asymptomatic cases are included [114]. Most episodes occur within the first months following the procedure [112].

The proposed mechanism for UEDVT associated with intracardiac devices is that transvenous leads, acting as foreign intravascular bodies, promote turbulent venous flow, which can lead to platelet aggregation and thrombosis [116]. Additionally, endothelial injury during implantation triggers an inflammatory response and activates the coagulation cascade, therefore inducing a prothrombotic state [117,118].

### 3.2. Clinical Manifestations

Most UEDVT cases associated with intracardiac devices are asymptomatic, although they can manifest with severe presentations, including SVCS or PE. When symptomatic, their symptoms are superimposable to those of other CRTs [112,116,119].

In the long term, as with DVT in other locations, PTS may develop [112]. Nevertheless, few studies report its occurrence in this specific type of patient, and its frequency remains unclear. In a series of 20 patients with venous thrombosis following permanent pacemaker implantation, Mandal et al. [116] reported that only 15% experienced occasional edema in the affected limb during follow-up. Conversely, a recent series of 12 patients with UEDVT associated with intracardiac devices found that half of the patients presented with signs or symptoms of PTS, with collateral circulation and edema being the most common [120].

Other long-term complications described in the literature include device dysfunction [121], esophageal varices due to bilateral subclavian thrombosis and SVCS [122], and a case of PM-associated phlegmasia cerulea dolens [123].

### 3.3. Risk Factors

Several factors may contribute to an increased risk of UEDVT associated with intracardiac devices, though they are not clearly defined. Regarding patients’ characteristics, older age and male sex appear to be associated with a higher risk [124,125,126], although some studies have not found this relationship [113,127,128,129]. A previous history of VTE has been linked to a 2-to-7-fold increase in risk [125,126,127,128,129,130]. Some studies suggest a protective role of antiplatelet therapy, showing a reduction of approximately two-thirds in thrombotic risk; however, this finding is not consistently supported [113,127,129,130]. Attempts to identify specific comorbidities, indications for device implantation, or types of devices associated with a higher thrombotic risk have yielded inconsistent results across studies [124,125,127,128,129].

Regarding device-specific factors, most studies agree that a higher number of leads increases thrombotic risk, with an OR ranging from 2 to 3.5 depending on the study [112,113,125,129,130]. This may be attributed to the larger total lead diameter, resulting in increased contact surface area between the endothelium and the foreign body [112]. A complicated or prolonged implantation procedure (exceeding 60 or 90 min, depending on the study) has also been associated with a heightened risk of UEDVT [127,128].

Other factors, such as lead material, site of insertion, or implantation side, have shown inconsistent impact on UEDVT risk, and current evidence is insufficient to confirm these associations [127,129]. Transient pacemakers seem to be more thrombogenic than permanent ones, likely due to greater stiffness and hardness, which may cause more significant vascular damage [128].

### 3.4. Management

The optimal treatment for these patients is not yet clearly established, and clinical guidelines do not specifically address them [120]. As mentioned previously, for CRT, the ESVS recommends anticoagulation for at least 3 months, with extended treatment if the catheter is not removed [88]. In the case of intracardiac devices, device removal is usually not feasible. Despite this, the duration of anticoagulation varies across healthcare centers and among physicians [88,124].

In this regard, Núñez Fernández et al. conducted a prospective study on UEDVT, including 156 patients with PM-related UEDVT. They found that the risk of thrombosis recurrence in these patients was higher than in those with UEDVT caused by other transient provoking factors, raising the question of whether the presence of an intracardiac device should be considered a transient or a permanent provoking factor in terms of treatment duration [124].

### 3.5. Prevention

Two studies have evaluated the benefits of prophylactic measures to prevent UEDVT after intracardiac device implantation. Costa et al. conducted a RCT involving 101 patients with cardiac device implantation and a left ventricular ejection fraction <40% and/or a history of the temporary pacing system. Participants were randomized to receive either placebo or warfarin (International Normalized Ratio (INR) goal of 2–3.5) for 6 months. Venography of the affected extremity revealed a relative risk of 0.63 for venous obstruction in the warfarin group. Only one of 49 patients in the warfarin group experienced gastrointestinal bleeding during treatment, with no deaths or pocket hematomas reported [131].

Seeger et al. [132] conducted a trial involving 20 patients who underwent PM implantation and received low doses of heparin for 14 days, compared to 20 patients who received no treatment post-implantation. Pulmonary scintigraphy performed after this period showed no perfusion defects in the heparin group, while three new cases of perfusion defects were identified in the control group.

**Figure 4:**
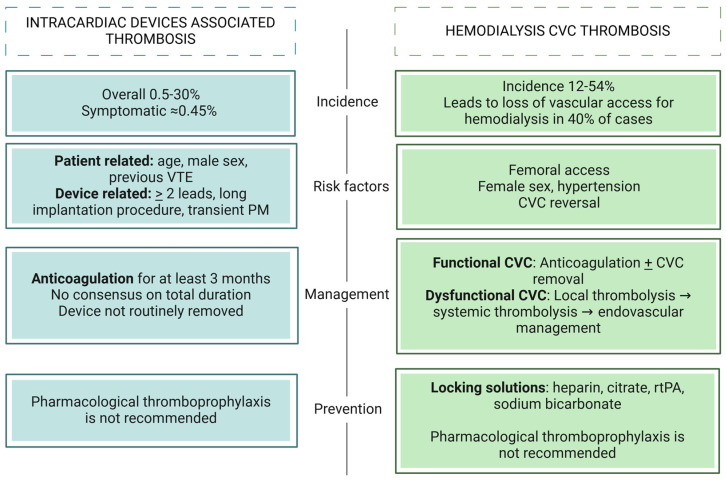
Overview of intracardiac devices- and hemodialysis CVC-associated thrombosis. VTE (venous thromboembolism), PM (pacemaker), CVC (central venous catheter), rtPA (recombinant tissue plasminogen activator).

Given the limited evidence, thrombosis prophylaxis is not routinely recommended for patients undergoing cardiac device implantation [88] (Figure 4).

## 4. Hemodialysis Accesses

There are several types of vascular access used for hemodialysis in patients with advanced chronic kidney disease (CKD), including arteriovenous fistulas (AVFs), synthetic arteriovenous grafts (AVGs), or tunneled CVCs [133]. Currently, the use of AVFs or AVGs is recommended over tunneled CVCs due to their lower rates of dysfunction. However, CVCs remain widely used, likely due to their ease of placement and the possibility of immediate hemodialysis initiation [134]. The most serious complications of CVCs are infection and catheter malfunction, often caused by CVC thrombosis. Recognizing thrombosis is critical, as it can result in the loss of vascular access for hemodialysis in up to 40% of cases [134,135].

### 4.1. Epidemiology and Pathophysiology

The incidence of hemodialysis CVC thrombosis varies between studies, ranging from 12% to 52%, likely due to different follow-up duration and different strategies in thrombosis prophylaxis [135,136,137,138].

As with other CVCs, thrombus formation is attributed to a combination of endothelial injury, which occurs both during insertion and due to the continued presence of a foreign body; activation of the coagulation cascade; and blood stasis during the interdialytic period [134,139]. Furthermore, the risk of thrombosis in these patients is exacerbated by CKD itself, which promotes VTE through activation of procoagulant factors, decreased levels of natural anticoagulants, increased platelet activation and aggregation, and reduced fibrinolytic activity [140].

### 4.2. Clinical Manifestations

Clinical manifestations of hemodialysis CVC thrombosis are similar to those of CRT, with catheter dysfunction being the most significant presentation due to its implications for patient care [134].

### 4.3. Risk Factors

The factors for CRT in hemodialysis patients have been infrequently studied or described in the literature. There is general agreement that femoral access is more prone to thrombosis and dysfunction than jugular access, likely due to the kinking occurring with hip flexion [141,142].

Mohazzab et al. conducted a prospective study involving 466 patients with CVCs for hemodialysis and identified a higher risk for thrombosis in females and hypertensive patients, while diabetes appeared to have a protective effect. They also observed an increased risk with age and body mass index, although these differences were not statistically significant [135]. Similarly, Ward et al. reported a tendency toward higher thrombosis risk in women. The only significant factor increasing thrombosis risk in their study was CVC reversal during one of the prior six hemodialysis sessions [143].

Premuzic et al. [144] identified a higher thrombosis risk when the catheter tip was positioned against the vein wall rather than centered in the lumen.

### 4.4. Management

Prompt management of hemodialysis CVC thrombosis is essential, as delays may lead to inadequate dialysis and increased morbidity. Three key factors must be considered: the availability of alternative vascular access, the usability of the CVC, and any contraindications to anticoagulation or thrombolysis [134].

When alternative vascular access is available, systemic anticoagulation (using non-fractioned heparin or LMWH followed by DOACs) should be initiated. Catheter removal is recommended, with a delay of 3 to 5 days if there is a high risk of embolization. Anticoagulation should be continued for at least 6 weeks after catheter removal [134].

When alternative access is unavailable or the CVC needs to be preserved, and it is partially occluded (allowing infusion but not blood extraction), local thrombolytic therapy via the CVC is indicated. Recombinant tissue plasminogen activators (rtPAs) such as alteplase or reteplase are preferred, as streptokinase carries a higher risk of anaphylaxis [134,139,142,145]. If local thrombolysis is ineffective, low-dose systemic thrombolysis may be attempted [134]. If catheter occlusion persists despite these measures, it is usually due to the formation of a fibrin sheath. Endovascular interventions, such as wire-guided catheter exchange or balloon dilation, are typically indicated [134,142].

In some cases, catheter removal is mandatory. These include thrombus infection, limb- or life-threatening thrombosis (CRAT, others), lack of response to anticoagulation, or contraindication to anticoagulation [134].

### 4.5. Prevention

Nowadays, the administration of locking solutions in the interdialytic period is the standardized method to prevent catheter dysfunction and thrombosis. The most common solutions are heparin, more frequently used in the United States, and trisodium citrate, more common in Europe [142]. Heparin is typically used at low concentrations, with potential adverse effects including heparin-induced thrombocytopenia or bleeding due to anticoagulant leakage to systemic circulation. Conversely, citrate prevents thrombosis through chelating calcium, thereby inhibiting the activation of calcium-dependent coagulation factors. Citrate is used at a low concentration (4%) to minimize the risk of symptomatic hypocalcemia, which can cause paresthesia, arrhythmias, and sudden death. Both locking solutions have been proven effective in preventing CVC thrombosis [134,139,142].

Hemmelgarn et al. conducted an RCT comparing the use of a locking solution three times per week with heparin twice a week, combined with an rtPA locking solution once a week. They found a lower incidence of CVC dysfunction in the group using the combined heparin and rtPA solutions [146]. Similarly, Islam et al. evaluated the use of sodium bicarbonate locking solution in non-tunneled hemodialysis catheters, comparing it to a heparin locking solution. They found no significant differences in malfunction or thrombosis rates [147].

Finally, some studies have explored the potential of systemic anticoagulation as prophylaxis for hemodialysis CVC thrombosis. Mokryzcki et al. [148] and Wilkieson et al. [149] compared warfarin at different doses (1 mg per day in the first study and an INR goal of 1.5–1.9 in the second) against placebo, finding no benefits from anticoagulation. In contrast, Abdul-Rahman et al. reported a lower incidence of CRT in hemodialysis patients receiving either warfarin (INR goal of 1.5–2) or aspirin (81 mg per day) compared to placebo. No bleeding events were reported; however, it is important to note that patients with a high bleeding risk were excluded from the trial [150].

Besides the insufficient evidence to recommend systemic anticoagulation for thrombosis prophylaxis, it is worth noting that CKD patients are at a high risk of bleeding, and the use of VKA may accelerate vascular calcification [141] (Figure 4).

## 5. Conclusions

With the increasing use of different vascular accesses, such as CVCs, PICCs, or implantable ports, it is essential to address one of their most common complications—CRT. Despite its clinical significance, many aspects remain unknown, such as its precise incidence or some of the risk factors associated with its occurrence. Although its management is somewhat standardized, several questions remain unanswered, including the optimal type of anticoagulant, the appropriate duration of anticoagulation based on patients’ characteristics, the indications for thrombolysis or endovascular approaches, and the potential benefit of pharmacologic thromboprophylaxis in selected patients.

In the specific case of CVCs for hemodialysis, effective management is essential to preserve vascular access for dialysis. However, the optimal prevention measures remain unclear, as CRT and catheter dysfunction still occur in a significant percentage of patients despite the use of locking solutions. Finally, thrombosis associated with intracardiac devices represents an area requiring further investigation, particularly regarding the optimal management and duration of anticoagulation therapy.

## Abbreviations

CRT	Catheter-related thrombosis
DVT	Deep vein thrombosis
PICC	Peripherally inserted central catheter
UEDVT	Upper extremity deep vein thrombosis
VTE	Venous thromboembolism
PE	Pulmonary embolism
SVCS	Superior Vena Cava syndrome
CVC	Central venous catheter
PM	Pacemaker
ICD	Implantable cardioverter-defibrillator
OR	Odds ratio
LEDVT	Lower extremity deep vein thrombosis.
CRAT	Catheter-related atrial thrombosis
PTS	Post-thrombotic syndrome
US	Ultrasonography
CTA	Computed tomography angiography
MRI	Magnetic resonance imaging
RCT	Randomized clinical trial
LMWH	Low-molecular-weight heparin
VKA	Vitamin K antagonist
ESVS	European Society of Vascular Surgery
DOAC	Direct oral anticoagulant
CDT	Catheter-directed thrombolysis
INR	International Normalized Ratio
CKD	Chronic kidney disease
AVF	Arteriovenous fistula
AVG	Arteriovenous graft
rtPA	Recombinant tissue plasminogen activator
